# The Effect of Electrical Stimulation–Induced Pain on Time Perception and Relationships to Pain-Related Emotional and Cognitive Factors: A Temporal Bisection Task and Questionnaire–Based Study

**DOI:** 10.3389/fpsyg.2021.800774

**Published:** 2022-01-14

**Authors:** Chun-Chun Weng, Ning Wang, Yu-Han Zhang, Jin-Yan Wang, Fei Luo

**Affiliations:** ^1^CAS Key Laboratory of Mental Health, Institute of Psychology, Chinese Academy of Sciences, Beijing, China; ^2^Department of Psychology, University of Chinese Academy of Sciences, Beijing, China

**Keywords:** time perception, interval timing, pain, electrical stimulation, emotion, cognition

## Abstract

Pain has not only sensory, but also emotional and cognitive, components. Some studies have explored the effect of pain on time perception, but the results remain controversial. Whether individual pain-related emotional and cognitive factors play roles in this process should also be explored. In this study, we investigated the effect of electrical stimulation–induced pain on interval timing using a temporal bisection task. During each task session, subjects received one of five types of stimulation randomly: no stimulus and 100 and 300 ms of non-painful and painful stimulation. Pain-related emotional and cognitive factors were measured using a series of questionnaires. The proportion of “long” judgments of a 1,200-ms visual stimulus duration was significantly smaller with 300 ms painful stimulation than with no stimulus (*P* < 0.0001) and 100 ms (*P* < 0.0001) and 300 ms (*P* = 0.021) non-painful stimulation. The point of subjective equality (PSE) did not differ among sessions, but the average Weber fraction (WF) was higher for painful sessions than for no-stimulus session (*P* = 0.022). The pain fear score correlated positively with the PSE under 100 ms non-painful (*P* = 0.031) and painful (*P* = 0.002) and 300 ms painful (*P* = 0.006) stimulation. Pain catastrophizing and pain anxiety scores correlated significantly with the WF under no stimulus (*P* = 0.005) and 100 ms non-painful stimulation (*P* = 0.027), respectively. These results suggest that electrical stimulation–induced pain affects temporal sensitivity, and that pain-related emotional and cognitive factors are associated with the processing of time perception.

## Introduction

Pain is defined by the International Association for the Study of Pain as “an unpleasant sensory and emotional experience associated with, or resembling that associated with, actual or potential tissue damage” (Raja et al., 2020). It has sensory and emotional components (Loeser and Treede, 2008), including pain-related fear, anxiety, and depression (Price, 2000). Some researchers have suggested that pain also has cognitive and social dimensions (Williams and Craig, 2016), inducing changes in attention, memory, and empathy (Eccleston and Crombez, 1999; Smith et al., 2021). The cognitive dimension of pain determines how individuals express and deal with pain (Williams et al., 2012). For example, some individuals facing pain tend to focus on and amplify its threat, engaging in pain-related catastrophic thinking (Sullivan et al., 1995), which can enhance the sensation of pain and is related closely to negative emotions such as fear and anxiety (Martel et al., 2016; Sugiura and Sugiura, 2016). The multidimensional nature of pain makes its impact on cognitive psychological processes, such as time perception, complex.

Patients with chronic pain commonly perceive the prolongation of time (Bilting et al., 1983; Zhang et al., 2012). In laboratory studies conducted with human subjects, cold pressor, thermal, and electrical forms of stimulation are used to explore the effect of pain on interval timing (Thorn and Hansell, 1993; Hellstrom and Carlsson, 1997; Khoshnejad et al., 2014; Ogden et al., 2015; Rey et al., 2017). However, the results obtained have been inconsistent. Some studies have shown that pain leads to the underestimation of temporal durations (Hellstrom and Carlsson, 1997; Khoshnejad et al., 2014), whereas others have shown that it leads to overestimation (Ogden et al., 2015; Rey et al., 2017). This inconsistency may be due to the diversity of time perception task paradigms, and/or to the multidimensional and complex nature of pain. Thus, exploration of the impact of pain on time perception should involve consideration not only of its sensory dimension, but also related emotional and cognitive factors.

Few studies have examined the effect of electrical stimulation–induced pain on time perception. Using a verbal estimation task, Piovesan et al. (2019) found that subjects significantly overestimated the duration of pain caused by high-intensity electrical stimulation. Compared with pain induced in the laboratory by other common means (e.g., thermal and cold stimuli), that induced by electrical stimulation may produce more cognitive and emotional changes affecting time perception. Sarigiannidis et al. (2017, 2020) noted the need to pay attention to the effects of electrical stimulation–related emotions in experiments conducted with this stimulus type. They found that anxiety, but not fear, induced by electrical stimulation caused the underestimation of time intervals (Sarigiannidis et al., 2017, 2020). Thus, the use of other research paradigms is needed to clarify the impact of electrical stimulation–induced pain on time perception and the roles of individual pain-related emotional and cognitive factors.

The purpose of this study was to explore whether pain caused by electrical stimulation affects individuals’ perception of the duration of neutral visual stimuli, and whether individual pain-related emotional and cognitive traits are related to this time perception. A temporal bisection task and a questionnaire-based survey, respectively, were used to investigate these research questions.

## Materials and Methods

### Subjects

In total, 30 students (10 males and 20 females, mean age 22.1 ± 0.4 years) recruited from universities near our institute participated in this study. Eligible subjects had normal or corrected-to-normal vision, no history of mental illness or chronic pain, no drug or alcohol abuse, no recent use of painkillers, and no recent injury affecting limb pain perception (e.g., leg injury). The volunteer participants were informed of the experimental procedure and provided written informed consent. After completing the experiment, they received a reward of 60 RMB. This study was approved by the Institutional Review Board of the Institute of Psychology, Chinese Academy of Sciences (no. H16036).

### Experimental Procedure

**FIGURE 1:**
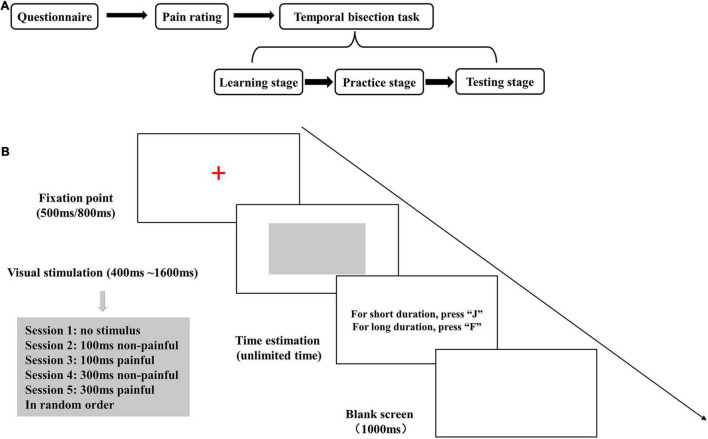
**(A)** Diagram of the study flow. **(B)** The experimental stage of the temporal bisection task. The gray rectangle was presented for 400, 600, 800, 1,000, 1,200, 1,400, or 1,600 ms. Electrical stimulation was applied during the last 100 or 300 ms of the visual stimulus presentation.

The subjects completed the whole experimental process in an independent laboratory in a single visit. They completed an electronic questionnaire, rated the intensity of painful and non-painful electrical stimuli, and performed a temporal bisection task with electrical stimulation (Figure 1A).

### Questionnaire

The electronic questionnaire-based survey included the Pain Catastrophizing Scale (PCS), Chinese Pain Anxiety Symptoms Scale (CHPASS), and Fear of Pain Questionnaire (FPQ) III. The PCS is used to assess the negative cognition affecting actual or expected pain, including redundant thinking, amplification, and helplessness, thereby reflecting individuals’ strategies for coping with pain (Sullivan et al., 1995; Darnall et al., 2017); it has 13 items rated on a 0–4 scale (total range, 0–52) (Yap et al., 2008). The CHPASS is used to measure pain-related anxiety; it has 20 items rated on a 0–5 scale (total range, 0–100) (McCracken and Dhingra, 2002; Wong et al., 2012). The FPQ is used to measure individuals’ fear of pain; it has 30 items rated on a 1–5 scale (total range, 30–150) (Osman et al., 2002). The Cronbach alpha values for the PCS, CHPASS, and FPQ in this study were 0.928, 0.915, and 0.940, respectively.

### Pain Rating

Electrical stimulation was applied to the subjects using a DS7A instrument (Digitimer Ltd., Glenwyn Garden City, United Kingdom). A pair of Ag/AgCl electrodes was attached 5 cm above the subject’s lateral malleolus. Each electrical stimulation pulse was an asymmetrical square wave applied for 2 ms; a 6-ms interstimulus interval was used (Van Ryckeghem et al., 2012) for the delivery of a total of 12 (100-ms session) or 36 (300-ms session) pulses.

The subjects rated the stimulation on a 0–10 scale (0 = no feeling; 1 = slight stimulation; 2 = obvious stimulation; 3 = very obvious stimulation, but no pain; 4 = slight pain; 5 = relatively painful; 6 = very painful; 7 = can’t bear more pain; 8 = extreme pain; 9 = feeling injured; 10 = unimaginable pain). Scores of 1–3 points were taken to indicate non-painful stimuli, scores of 4–7 points were considered to reflect tolerable pain under laboratory conditions, and scores of 8–10 points were taken to indicate intolerable pain (Gong et al., 2020).

The subjects first underwent 300 ms electrical stimulation at an initial current intensity of 0.5 mA, which was increased by 0.1 mA until the participant reported a score of 6 or was unwilling to receive a more intense stimulus. The current intensities at scores of 2, 4, 5, and 6 were recorded. The maximum intensity of those generating multiple reports of the same pain score was taken as the current intensity for that score. When a subject reported pain at the initial current intensity of 0.5 mA, this intensity was adjusted to 0.1 mA and then increased in 0.02- or 0.01-mA increments. Then, we took the current intensity generating a pain score of 2 or 5 with 300 ms electrical stimulation as the initial intensity for 100 ms electrical stimulation, and adjusted it slightly according to the actual situation to ensure that the score was also 2 or 5 at 100 ms electrical stimulation.

### Temporal Bisection Task

The E-Prime 1.2 software (Psychology Software Tools, Pittsburg, PA, United States) was used for programming and data acquisition for the temporal bisection task. The task was performed with a 12.1-inch color monitor with a resolution of 1,280 × 800 pixels, a refresh rate of 60 Hz, and a white display background. In each trial, an initial fix-point (a red “+”) was presented for 500- or 800-ms (randomly selected), and then a gray rectangle was displayed in the center of the screen as the neutral visual stimulus. The subjects rated the neutral stimulus presentation as “long” or “short” by pressing the “F” or “J” key (balanced between subjects). After rating, a blank screen was displayed as a buffer for 1,000 ms, and then the next trial began (Figure 1B).

The temporal bisection task was adopted from previous studies (Fayolle and Droit-Volet, 2014; Huang et al., 2018) and performed in three stages. In the learning stage, the gray rectangle was displayed five times each for 400 ms (standard short duration) and 1,600 ms (standard long duration), in random order. The subjects were asked to remember these two durations, and no timing strategy was used (Matthews and Meck, 2016).

In the practice stage, the two standard durations were presented three times each in random order, the subjects rated them, and feedback on the correctness of their responses was displayed on the screen. Then, standard- and intermediate-duration (600-, 800-, 1, 200-, and 1,400-ms) stimuli were presented twice each in random order, and the subjects rated whether the stimulus durations were closer to “long” or “short.” Instead of feedback after each rating, the total accuracy was presented after the completion of the exercise. Subjects repeated the learning and practice stages until they attained >70% accuracy, upon which they proceeded to the testing stage.

In the testing stage, seven standard- and intermediate-duration (400-, 600-, 800-, 1, 000-, 1, 200-, 1, 400-, and 1,600-ms) gray rectangle stimuli were presented 10 times each in random order per session. The subjects rated stimulus duration without feedback. The experiment consisted of five sessions conducted with no electrical stimulation (no stimulus) and with electrical stimulation at the pain scores of 2 (non-painful) and 5 (painful) during the last 100 and 300 ms of visual stimulation, respectively. The five sessions were administered in random order while avoiding two consecutive painful sessions.

### Statistical Analysis

Prism 8 (GraphPad Software, Inc., La Jolla, CA, United States) was used for the statistical analysis. Following previous studies (Ward and Odum, 2007; Deane et al., 2017), the proportion of “long” responses (P_L_) for each visual stimulus duration was recorded to analyze time perception using a fitting curve:


$$F\,\left(t\right)=a+\frac{b}{\sigma \,\sqrt{2\,\pi }}\,\int_{-\infty }^{t}\left[\exp-\left(\frac{{\left(t-\mu \right)}^{2}}{2\,\sigma^{2}}\right)\right]\,dt$$

In this function, *F*(*t*) is the P_L_ at *t* duration. The point of subjective equality (PSE) is the mean (μ) of this function, and the Weber fraction (WF) is equal to the standard deviation (σ) divided by the PSE. The PSE represents the subjectively perceived length of time, and an increase in the WF reflects a decrease in temporal sensitivity. The results are presented as means ± standard errors of the mean. Student’s *t* test and one- and two-way repeated-measures analyses of variance (RM ANOVAs) were used to compare the current intensities, P_L_s for each duration, PSEs, and WFs among sessions. Bonferroni analysis was used for *post-hoc* testing. Pearson’s correlations were used to assess relationships among questionnaire results, stimulus intensity, and temporal bisection task performance. Multivariate linear regression was also performed to assess factors associated with time perception (see Supplementary Table 1 for details). *P* < 0.05 was set as the significance level.

## Results

### Electrical Stimulation Intensities and Questionnaire Scores

The mean current intensity for the 100- and 300-ms non-painful sessions was 0.77 ± 0.07 mA. The mean intensities for 100- and 300-ms painful sessions were 2.07 ± 0.20 and 1.94 ± 0.18 mA, respectively. The average current intensity for all painful sessions (2.01 ± 0.18 mA) was significantly higher than that for non-painful sessions [*t*_(29)_ = 8.390, *P* < 0.0001]. The average PCS, CHPASS, and FPQ scores were 20.80 ± 1.77, 44.77 ± 2.88, and 100.40 ± 3.03, respectively.

### Temporal Bisection Task Performance

**FIGURE 2:**
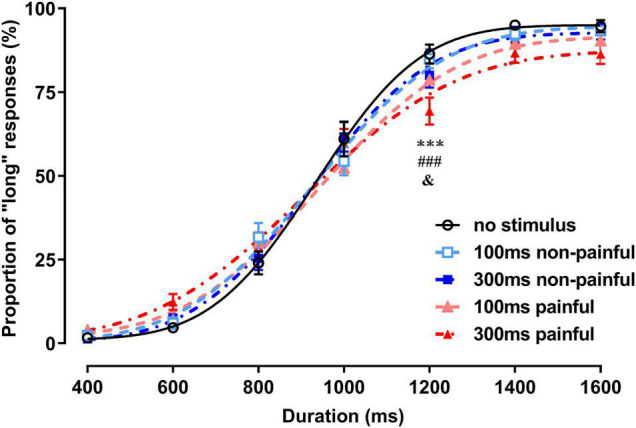
Proportions of long responses and fitting curves for the five sessions of the temporal bisection task. ***P < 0.001, 300-ms painful vs. no stimulus; ###*P* < 0.001, 300-ms painful vs. 100-ms non-painful; &*P* < 0.05, 300-ms painful vs. 300-ms non-painful.

In the P_L_ analysis, the main effect of the duration was significant [*F*_(6_,_174)_ = 514.8, *P* < 0.0001, $\eta_{p}^{2}$ = 0.947], indicating that the subjects effectively distinguished the visual stimulus durations (Figure 2). The main effect of the session was not significant, but the interaction between the duration and session was [*F*_(24_,_696)_ = 3.65, *P* < 0.0001, $\eta_{p}^{2}$ = 0.112]. *Post-hoc* Bonferroni analysis showed that the P_L_ for the 300-ms painful session was significantly smaller than those for the no-stimulus and 100-ms and 300-ms non-painful sessions with 1,200-ms duration (*P* < 0.0001, *P* < 0.0001, and *P* = 0.021, respectively).

**FIGURE 3:**
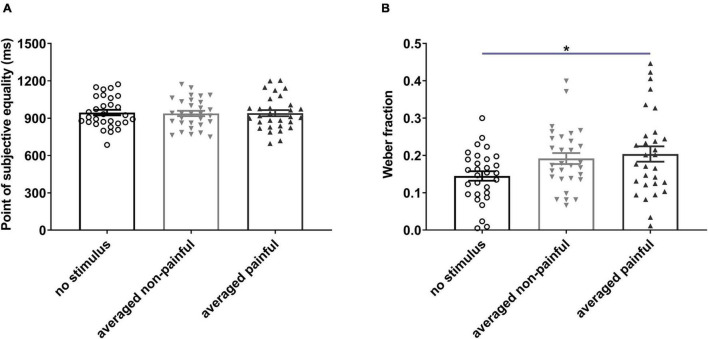
Points of subjective equality **(A)** and Weber fractions **(B)** under different electrical stimulation conditions. Averaged non-painful: averaged PSE or WF of 100- and 300-ms non-painful sessions; Averaged painful: averaged PSE or WF of 100- and 300-ms painful sessions. *P < 0.05, averaged painful vs. no stimulus.

One-way RM ANOVA revealed significant differences in the WF among the no-stimulus, non-painful, and painful states [*F*_(2,58)_ = 4.297, *P* = 0.0182, $\eta_{p}^{2}$ = 0.129; Figure 3B]. The average WF was significantly higher for painful sessions than for no-stimulus session (*P* = 0.022), suggesting that electrical stimulation reduced temporal sensitivity. No significant difference in the PSE was observed among the three states (Figure 3A). In addition, the PSE and WF did not differ among the five sessions (see Supplementary Presentation 1).

### Relationships Among Questionnaire Results, Stimulus Intensity, and Time Perception

The results of the correlation analysis are presented in Table 1. The intensities of non-painful and painful stimulation correlated positively (*r* = 0.677, *P* < 0.0001), suggesting that subjects’ sensitivity to these stimulation types was consistent. The PSEs for the no-stimulus and 300-ms non-painful and painful sessions correlated positively with the average intensity of painful stimulation (*r* = 0.522, *P* = 0.003; *r* = 0.381, *P* = 0.038; and *r* = 0.494, *P* = 0.006, respectively), reflecting an increase in the PSE with the current intensity. The PSEs for the 100-ms non-painful and 100- and 300-ms painful sessions correlated negatively with the FPQ score (*r* = –0.396, *P* = 0.031; *r* = –0.537, *P* = 0.002; and *r* = –0.489, *P* = 0.006, respectively), suggesting that subjects with strong fear of pain tend to have lower PSEs.

**TABLE 1:** Coefficients of correlation (*r*) among stimulus intensity, pain-related scale scores, and temporal bisection results.

	Intensity		PCS	CHPASS	FPQ
	Non-painful	Averaged painful			
Intensity					
Averaged painful	0.677***				
Questionnaires					
PCS	−0.436*	−0.447*			
CHPASS	−0.435*	−0.449*	0.878***		
FPQ	–0.303	–0.199	0.267	0.379*	
PSE					
No stimulus	0.359	0.522**	–0.175	–0.297	–0.236
100-ms non-painful	0.250	0.148	0.115	–0.030	−0.396*
100-ms painful	0.086	0.211	–0.096	–0.197	−0.537**
300-ms non-painful	0.298	0.381*	–0.035	–0.213	–0.224
300-ms painful	0.375*	0.494**	–0.157	–0.322	−0.489**
WF					
No stimulus	0.249	0.130	−0.495**	–0.311	–0.212
100-ms non-painful	0.094	–0.025	–0.343	−0.405*	0.214
100-ms painful	–0.191	–0.089	0.175	0.294	0.017
300-ms non-painful	–0.275	–0.225	0.056	0.029	0.255
300-ms painful	–0.069	–0.171	0.002	0.088	0.079

PCS, Pain Catastrophizing Scale; CHPASS, Chinese Pain Anxiety Symptoms Scale; FPQ, Fear of Pain Questionnaire III; PSE, point of subjective equality; WF, Weber fraction. *P < 0.05, **P < 0.01, and ***P < 0.001. The bold values represent significant correlation.

The PCS and CHPASS scores correlated negatively with the non-painful (*r* = –0.436, *P* = 0.0161 and *r* = –0.435, *P* = 0.0164, respectively) and painful (*r* = –0.447, *P* = 0.0134 and *r* = –0.449, *P* = 0.0129, respectively) current intensities, suggesting that subjects with stronger pain-catastrophizing cognition and pain anxiety tend to have lower sensory and pain thresholds. In addition, the PCS score correlated negatively with the WF for no-stimulus sessions (*r* = –0.495, *P* = 0.005), and the CHPASS score correlated negatively with the WF for 100-ms non-painful sessions (*r* = –0.405, *P* = 0.027), reflecting associations of pain-catastrophizing cognition and pain anxiety with time sensitivity. The CHPASS score correlated positively with the PCS (*r* = 0.878, *P <* 0.001) and FPQ (*r* = 0.379, *P* = 0.039) scores, suggesting that individuals with stronger pain-related anxiety also have stronger pain-related catastrophic thinking and fear.

The multivariate linear regression analysis showed that the interaction between the session type and FPQ score was the only significantly predictor of PSEs [Pillai’s *V* = 0.56, *F*_(4,19)_ = 6.15, *P* = 0.002, $\eta_{p}^{2}$ = 0.56]. Closer inspection showed that the FPQ score significantly predicted the PSE for the 100-ms painful session (*B* = –5.39, β = –0.57, *t* = –2.97, *P* = 0.007) and 300-ms painful session (*B* = –3.48, β = –0.42, *t* = –2.40, *P* = 0.025). Besides, averaged painful intensity significantly predicted the PSE for the 300-ms painful session (*B* = 70.03, β = 0.53, *t* = –2.38, *P* = 0.027). Other factors had no predictive effect on the PSE, and no factor was predictive of the WF (see Supplementary Table 1).

## Discussion

This study showed that subjects receiving painful electrical stimulation had reduced temporal sensitivity and underestimated the duration of 1,200-ms neutral visual stimuli. Pain-related fear scores correlated negatively with PSEs for non-painful and painful sessions. In addition, pain-related catastrophizing and anxiety scores correlated negatively with WFs for no-stimulus and 100-ms non-painful sessions. These results confirm the effect of electrical stimulation–induced pain on time perception, and suggest that pain-related emotional and cognitive factors are involved in the processing thereof.

Pain induced by electrical stimulation may attract attention, resulting in the weakening of attention resources allocated to the estimation of neutral visual stimulus duration; this factor may explain the weakening of temporal sensitivity and underestimation of the 1,200-ms duration during 300-ms painful sessions observed in this study. Pain indicates potential danger and can preferentially capture attention resources (Baliki and Apkarian, 2015), a phenomenon termed “attentional bias to pain” (Crombez et al., 2015). Zakay and Block (1995) proposed that attention plays a gating role in the processing of time estimation. Many studies have also confirmed that when more resources are allocated to other events, the resources involved in the internal clock will be reduced accordingly, resulting in the reduction of timing accuracy (Buhusi and Meck, 2009).

Another important factor affecting the processing of time perception is arousal (Gil and Droit-Volet, 2012; Yoo and Lee, 2015). High arousal levels can increase the pacemaker pulse rate, leading to the overestimation of time intervals. As a very important physiological phenomenon for survival, arousal to avoid danger can be caused by acute pain. Changes in alertness and attention caused by pain may jointly affect time perception. In this study, we found that pain induced by electrical stimulation reduced subjects’ time sensitivity (increased WFs) and led to slight underestimation of stimulus durations, suggesting that the distraction caused by electrical stimulation–induced pain had a greater impact on time perception than did pain-related arousal.

The PSE is an important index of subjective time estimation. In this study, it correlated negatively with the FPQ score in the 300-ms painful and 100-ms painful and non-painful sessions, meaning that individuals with higher pain-related fear scores had smaller PSE values reflecting the overestimation of neutral visual stimulus duration. In the regression analysis, the FPQ score was associated with the PSE for the 100-ms and 300-ms painful session. Many researchers investigating the impact of fear on time perception have reached conclusions consistent with these findings. For example, Brown et al. (2007) found that rats’ fear of foot shock led to the overestimation of time intervals. Such overestimation has also been observed with human subjects’ viewing of short horror films (Martinez-Rodrigo et al., 2020) and fear-inducing pictures (Grommet et al., 2019). According to the scalar timing theory (Gibbon et al., 1984), arousal caused by fear accelerates the processing of time perception, resulting in duration overestimation. This study confirmed that this phenomenon is also associated with pain-related fear state. The correlation of pain-related fear scores with time perception not only in painful sessions, but also in non-painful session, suggests that pain-related emotional components affect time perception in the absence of pain perception.

In this study, we used the Pain Catastrophizing Scale to explore individuals’ negative thinking. The PCS score correlated negatively with the WF in no-stimulus sessions, suggesting that individuals with more pain catastrophizing usually have greater time sensitivity. One possible explanation for this association is that individuals with higher PCS scores amplify the threat of potential pain, thereby enhancing their arousal to this threat specifically and the environment in general. The results of this study confirm the association of pain-related cognitive factors with time perception.

Another possible explanation for the study findings is that pain-related negative cognition indirectly affects time perception by affecting pain-related emotions and pain perception. The positive correlation between PCS and CHPASS scores in this study is consistent with previous findings (Lee et al., 2013) and is well understood, as stronger catastrophizing about pain may lead to greater pain-related anxiety. In addition, we found that PCS and CHPASS scores correlated negatively with the current intensity, suggesting that higher levels of pain-related emotions and cognition render subjects more sensitive to pain, reducing the current intensity required to achieve the same pain score. The effects of pain-related catastrophic thinking and anxiety on pain sensitivity have been demonstrated in many studies (Failla et al., 2020; Grouper et al., 2021; Kim et al., 2021). Taken together, the significant correlations observed in this study between the current intensity and the PSE and PCS and CHPASS scores suggest that the impacts of pain-related cognitive, emotional, and sensory factors on time perception may be direct and/or indirect.

### Study Limitations

In this study, the order of the five sessions was random and the subjects knew that they would feel electrical stimulation–induced pain at some point. Thus, they were likely to be anticipating such pain (i.e., be affected by pain-related emotional and cognitive factors) during the no-stimulus and non-painful sessions. This factor may have reduced differences among sessions in the effects of pain on time perception. However, a main focus of this study was to determine whether pain-related emotional and cognitive traits were associated with time perception in the absence of pain perception. In addition, the randomization of the session order helped to balance the learning effect caused by the repeated execution of temporal bisection tasks. Another limitation is that we do not assess pain-related emotion and cognition after each session, which prevented us from detecting between-session differences therein and their effects on time perception. Despite these shortcomings, however, this study demonstrated that individuals’ pain-related fear, anxiety, and disastrous thinking patterns are related significantly to (and may affect the processing of) time perception. The use of more sophisticated study designs in future research will help to reveal in greater detail the impacts of multiple dimensions of pain on time perception and the mechanisms underlying these effects.

## Conclusion

The results of this study show that electrical stimulation–induced pain can reduce temporal sensitivity with no obvious effect on subjective perception. Furthermore, pain-related emotional and cognitive factors have potential effects on time perception at the individual level. This study revealed close correlations between pain-related fear and subjective time estimation, as well as between pain coping strategies and pain-related anxiety and time sensitivity. These results suggest that more attention needs to be paid to individuals’ regulation of pain-related emotional and cognitive factors when exploring the impact of pain on time perception. The findings of this study provide important clues for further research of this nature.

## Data Availability Statement

The raw data supporting the conclusions of this article will be made available by the authors, without undue reservation.

## Ethics Statement

The studies involving human participants were reviewed and approved by the Institutional Review Board of the Institute of Psychology, Chinese Academy of Sciences. The patients/participants provided their written informed consent to participate in this study.

## Author Contributions

NW, J-YW, and FL contributed to the conception and design of the study. C-CW performed the experiment and the statistical analysis. C-CW and NW wrote the first draft of the manuscript. Y-HZ revised the manuscript. All authors contributed to manuscript revision, read, and approved the submitted version.

## Conflict of Interest

The authors declare that the research was conducted in the absence of any commercial or financial relationships that could be construed as a potential conflict of interest.

## Publisher’s Note

All claims expressed in this article are solely those of the authors and do not necessarily represent those of their affiliated organizations, or those of the publisher, the editors and the reviewers. Any product that may be evaluated in this article, or claim that may be made by its manufacturer, is not guaranteed or endorsed by the publisher.
