# Supplementary material for: The Effect of Electrical Stimulation–Induced Pain on Time Perception and Relationships to Pain-Related Emotional and Cognitive Factors: A Temporal Bisection Task and Questionnaire–Based Study
Source: Front Psychol. 2022 Jan 14;12:800774. doi: 10.3389/fpsyg.2021.800774 (PMC8795068; doi:10.3389/fpsyg.2021.800774)
Supplement: Supplementary file 1 [file Table_1.DOCX]

To control for the potential confounding effects of gender, age and other factors, we conducted two multivariate linear regression analyses for PSE and WF, respectively, using IBM SPSS 26.0 with the following codes:

[GLM PSE1 PSE2 PSE3 PSE4 PSE5 WITH Gender year nopain pain PCS CHPASS FPQ_III

/WSFACTOR=session 5 Polynomial

/METHOD=SSTYPE(3)

/PRINT=PARAMETER

/CRITERIA=ALPHA(.05)

/WSDESIGN=session

/DESIGN=Gender year nopain pain PCS CHPASS FPQ_III.]

[GLM WF1 WF2 WF3 WF4 WF5 WITH Gender year nopain pain PCS CHPASS FPQ_III

/WSFACTOR= session 5 Polynomial

/METHOD=SSTYPE(3)

/PRINT=PARAMETER

/CRITERIA=ALPHA(.05)

/WSDESIGN= session

/DESIGN=Gender year nopain pain PCS CHPASS FPQ_III.]

In these codes, “nopain” means the intensity of non-painful stimuli and “pain” means the intensity of averaged painful stimuli. Besides, PSE1-PSE5, WF1-WF5 represents the PSEs and WFs in five sessions

PSE1, PSE of no-stimulus session; PSE2, PSE of 100-ms non-painful session; PSE3, PSE of 100-ms painful session, PSE4, PSE of 300-ms non-painful session; PSE5, PSE of 300-ms painful session.

WF1, WF of no-stimulus session; WF2, WF of 100-ms non-painful session; WF3, WF of 100-ms painful session, WF4, WF of 300-ms non-painful session; WF5, WF of 300-ms painful session.

**Supplementary Table 1 Multivariate Tests of PSE**

| Effect | Pillai’s V | F | Hypothesis df | Error df | Sig. |
| --- | --- | --- | --- | --- | --- |
| session | .337 | 2.417 | 4.000 | 19.000 | .084 |
| session * Gender | .310 | 2.134 | 4.000 | 19.000 | .116 |
| session * year | .043 | .215 | 4.000 | 19.000 | .927 |
| session * nopain | .165 | .939 | 4.000 | 19.000 | .463 |
| session * pain | .274 | 1.792 | 4.000 | 19.000 | .172 |
| session * PCS | .231 | 1.428 | 4.000 | 19.000 | .263 |
| session * CHPASS | .283 | 1.878 | 4.000 | 19.000 | .156 |
| session * FPQ_III | .564 | 6.152 | 4.000 | 19.000 | .002 |

Design: Intercept + Gender + year + nopain + pain + PCS + CHPASS + FPQ_III

Within Subjects Design: session

**Supplementary Table 2** Parameter Estimates of PSE

| Dependent Variable | Parameter | B | Std. Error | β | t | Sig. | 95% Confidence Interval | |
| --- | --- | --- | --- | --- | --- | --- | --- | --- |
|  |  |  |  |  |  |  | Lower Bound | Upper Bound |
| PSE1 | Intercept | 1012.261 | 249.061 |  | 4.064 | .001 | 495.740 | 1528.782 |
|  | Gender | 70.873 | 57.082 | .275 | 1.242 | .227 | -47.508 | 189.253 |
|  | year | -5.932 | 9.114 | -.118 | -.651 | .522 | -24.832 | 12.968 |
|  | nopain | -21.678 | 81.542 | -.065 | -.266 | .793 | -190.786 | 147.430 |
|  | pain | 49.498 | 30.680 | .405 | 1.613 | .121 | -14.129 | 113.126 |
|  | PCS | 5.369 | 4.719 | .421 | 1.138 | .268 | -4.419 | 15.156 |
|  | CHPASS | -3.345 | 2.921 | -.428 | -1.145 | .264 | -9.404 | 2.713 |
|  | FPQ_III | -.043 | 1.466 | -.006 | -.029 | .977 | -3.084 | 2.998 |
| PSE2 | Intercept | 1309.840 | 247.031 |  | 5.302 | .000 | 797.529 | 1822.152 |
|  | Gender | 88.998 | 56.617 | .348 | 1.572 | .130 | -28.417 | 206.414 |
|  | year | -13.060 | 9.039 | -.261 | -1.445 | .163 | -31.806 | 5.686 |
|  | nopain | 62.294 | 80.878 | .186 | .770 | .449 | -105.436 | 230.024 |
|  | pain | -14.952 | 30.430 | -.123 | -.491 | .628 | -78.060 | 48.157 |
|  | PCS | 5.895 | 4.681 | .465 | 1.259 | .221 | -3.812 | 15.603 |
|  | CHPASS | -1.746 | 2.898 | -.224 | -.602 | .553 | -7.755 | 4.264 |
|  | FPQ_III | -1.767 | 1.455 | -.239 | -1.215 | .237 | -4.784 | 1.250 |
| PSE3 | Intercept | 1535.294 | 307.760 |  | 4.989 | .000 | 897.038 | 2173.550 |
|  | Gender | -55.009 | 70.535 | -.175 | -.780 | .444 | -201.290 | 91.272 |
|  | year | -2.964 | 11.261 | -.048 | -.263 | .795 | -26.319 | 20.391 |
|  | nopain | -87.203 | 100.760 | -.213 | -.865 | .396 | -296.167 | 121.761 |
|  | pain | 52.412 | 37.911 | .351 | 1.383 | .181 | -26.211 | 131.035 |
|  | PCS | 2.261 | 5.832 | .145 | .388 | .702 | -9.834 | 14.355 |
|  | CHPASS | -.927 | 3.610 | -.097 | -.257 | .800 | -8.414 | 6.559 |
|  | FPQ_III | -5.386 | 1.812 | -.595 | -2.972 | .007 | -9.145 | -1.628 |
| PSE4 | Intercept | 1063.785 | 268.605 |  | 3.960 | .001 | 506.733 | 1620.837 |
|  | Gender | 44.570 | 61.561 | .170 | .724 | .477 | -83.100 | 172.240 |
|  | year | -7.617 | 9.829 | -.148 | -.775 | .447 | -28.001 | 12.766 |
|  | nopain | 17.835 | 87.941 | .052 | .203 | .841 | -164.543 | 200.213 |
|  | pain | 33.775 | 33.088 | .270 | 1.021 | .318 | -34.845 | 102.395 |
|  | PCS | 8.751 | 5.090 | .672 | 1.719 | .100 | -1.805 | 19.306 |
|  | CHPASS | -4.830 | 3.151 | -.604 | -1.533 | .140 | -11.364 | 1.704 |
|  | FPQ_III | -.173 | 1.582 | -.023 | -.109 | .914 | -3.453 | 3.107 |
| PSE5 | Intercept | 1260.751 | 246.243 |  | 5.120 | .000 | 750.075 | 1771.428 |
|  | Gender | -68.254 | 56.436 | -.239 | -1.209 | .239 | -185.295 | 48.787 |
|  | year | -4.719 | 9.010 | -.084 | -.524 | .606 | -23.406 | 13.967 |
|  | nopain | 20.190 | 80.619 | .054 | .250 | .805 | -147.004 | 187.385 |
|  | pain | 72.030 | 30.333 | .530 | 2.375 | .027 | 9.123 | 134.937 |
|  | PCS | 6.819 | 4.666 | .481 | 1.461 | .158 | -2.858 | 16.496 |
|  | CHPASS | -3.560 | 2.888 | -.410 | -1.233 | .231 | -9.550 | 2.430 |
|  | FPQ_III | -3.484 | 1.450 | -.422 | -2.403 | .025 | -6.490 | -.477 |

**Supplementary Table 3** Multivariate Tests of Weber fraction

| Effect | Pillai’s V | F | Hypothesis df | Error df | Sig. |
| --- | --- | --- | --- | --- | --- |
| session | .068 | .346 | 4.000 | 19.000 | .844 |
| session * Gender | .063 | .318 | 4.000 | 19.000 | .862 |
| session * year | .010 | .048 | 4.000 | 19.000 | .995 |
| session * nopain | .107 | .569 | 4.000 | 19.000 | .688 |
| session * pain | .062 | .314 | 4.000 | 19.000 | .865 |
| session * PCS | .197 | 1.167 | 4.000 | 19.000 | .356 |
| session * CHPASS | .283 | 1.875 | 4.000 | 19.000 | .156 |
| session * FPQ_III | .145 | .807 | 4.000 | 19.000 | .536 |

Design: Intercept + Gender + year + nopain + pain + PCS + CHPASS + FPQ_III

Within Subjects Design: session

**Supplementary Table 4** Parameter Estimates of WF

| Dependent Variable | Parameter | B | Std. Error | β | t | Sig. | 95% Confidence Interval | |
| --- | --- | --- | --- | --- | --- | --- | --- | --- |
|  |  |  |  |  |  |  | Lower Bound | Upper Bound |
| WF1 | Intercept | .153 | .130 |  | 1.171 | .254 | -.118 | .423 |
|  | Gender | -.059 | .030 | -.406 | -1.967 | .062 | -.121 | .003 |
|  | year | .006 | .005 | .202 | 1.200 | .243 | -.004 | .016 |
|  | nopain | .042 | .043 | .222 | .984 | .336 | -.047 | .130 |
|  | pain | .000 | .016 | .002 | .010 | .992 | -.033 | .033 |
|  | PCS | -.007 | .002 | -.945 | -2.742 | .012 | -.012 | -.002 |
|  | CHPASS | .003 | .002 | .609 | 1.753 | .094 | .000 | .006 |
|  | FPQ_III | -.001 | .001 | -.302 | -1.642 | .115 | -.003 | .000 |
| WF2 | Intercept | .346 | .222 |  | 1.559 | .133 | -.114 | .807 |
|  | Gender | -.091 | .051 | -.417 | -1.793 | .087 | -.197 | .014 |
|  | year | .006 | .008 | .141 | .742 | .466 | -.011 | .023 |
|  | nopain | .043 | .073 | .151 | .594 | .558 | -.108 | .194 |
|  | pain | -.014 | .027 | -.135 | -.513 | .613 | -.071 | .043 |
|  | PCS | .000 | .004 | .026 | .067 | .947 | -.008 | .009 |
|  | CHPASS | -.003 | .003 | -.477 | -1.220 | .236 | -.009 | .002 |
|  | FPQ_III | -.001 | .001 | -.197 | -.954 | .350 | -.004 | .001 |
| WF3 | Intercept | .089 | .248 |  | .360 | .722 | -.425 | .604 |
|  | Gender | -.014 | .057 | -.061 | -.238 | .814 | -.131 | .104 |
|  | year | .006 | .009 | .129 | .613 | .546 | -.013 | .024 |
|  | nopain | -.067 | .081 | -.233 | -.826 | .418 | -.235 | .101 |
|  | pain | .022 | .031 | .207 | .712 | .484 | -.042 | .085 |
|  | PCS | -.004 | .005 | -.362 | -.843 | .408 | -.014 | .006 |
|  | CHPASS | .005 | .003 | .671 | 1.548 | .136 | -.002 | .011 |
|  | FPQ_III | -.001 | .001 | -.208 | -.907 | .374 | -.004 | .002 |
| WF4 | Intercept | .008 | .253 |  | .033 | .974 | -.516 | .532 |
|  | Gender | -.047 | .058 | -.206 | -.810 | .427 | -.167 | .073 |
|  | year | .008 | .009 | .188 | .904 | .376 | -.011 | .028 |
|  | nopain | -.049 | .083 | -.165 | -.590 | .561 | -.220 | .123 |
|  | pain | -.002 | .031 | -.023 | -.079 | .938 | -.067 | .062 |
|  | PCS | .002 | .005 | .217 | .510 | .615 | -.007 | .012 |
|  | CHPASS | -.003 | .003 | -.366 | -.854 | .402 | -.009 | .004 |
|  | FPQ_III | .001 | .001 | .180 | .793 | .436 | -.002 | .004 |
| WF5 | Intercept | .061 | .411 |  | .147 | .884 | -.793 | .914 |
|  | Gender | -.083 | .094 | -.236 | -.881 | .388 | -.279 | .112 |
|  | year | .009 | .015 | .133 | .609 | .549 | -.022 | .040 |
|  | nopain | .059 | .135 | .128 | .435 | .668 | -.221 | .338 |
|  | pain | -.022 | .051 | -.132 | -.436 | .667 | -.127 | .083 |
|  | PCS | -.005 | .008 | -.299 | -.667 | .511 | -.021 | .011 |
|  | CHPASS | .003 | .005 | .295 | .652 | .521 | -.007 | .013 |
|  | FPQ_III | .000 | .002 | -.046 | -.195 | .847 | -.005 | .005 |
