# Supplementary material for: The Effect of Electrical Stimulation–Induced Pain on Time Perception and Relationships to Pain-Related Emotional and Cognitive Factors: A Temporal Bisection Task and Questionnaire–Based Study
Source: Front Psychol. 2022 Jan 14;12:800774. doi: 10.3389/fpsyg.2021.800774 (PMC8795068; doi:10.3389/fpsyg.2021.800774)
Supplement: Supplementary file 2 [file Presentation_1.PPTX]

## Slide 1
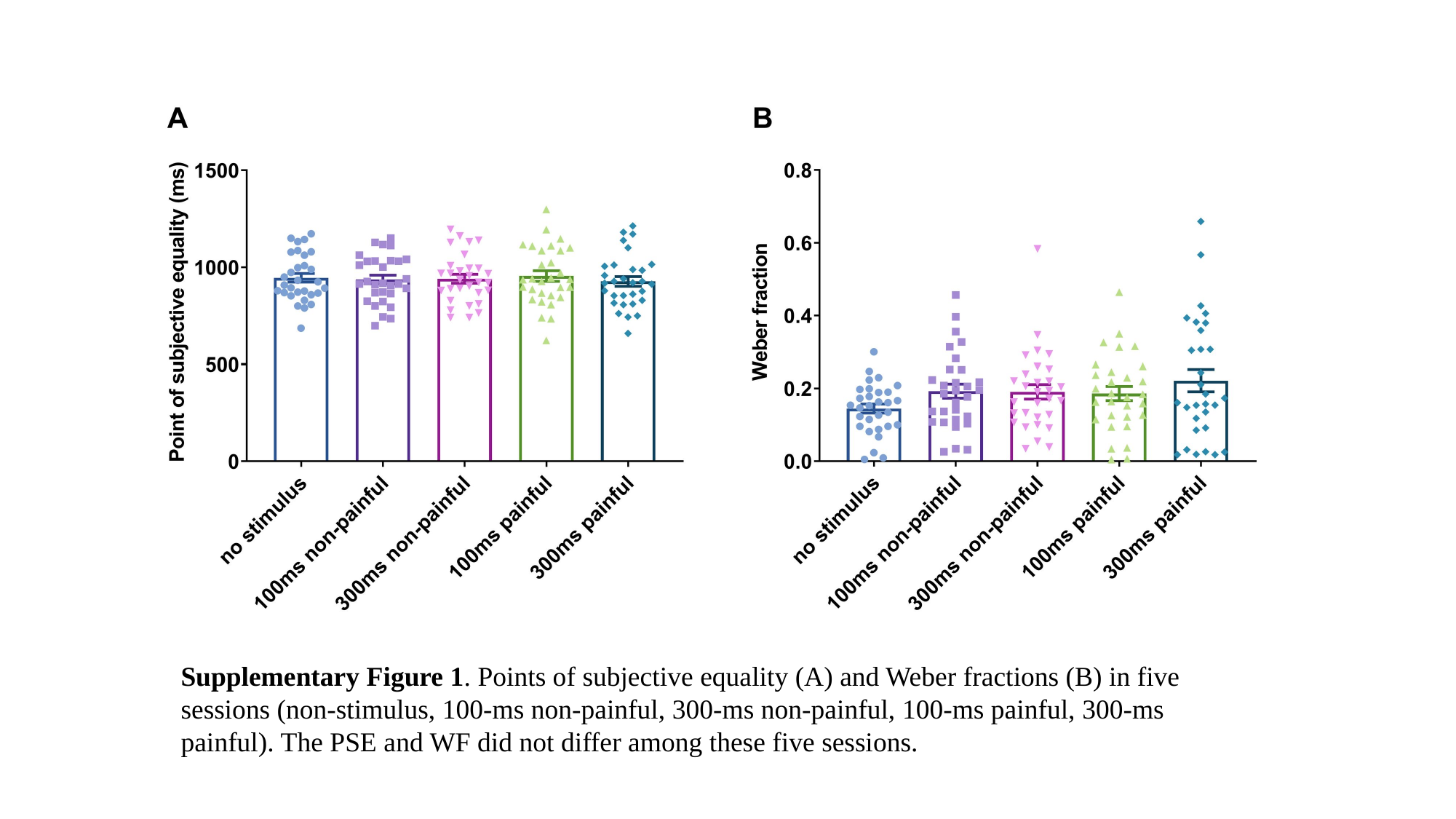

Supplementary Figure 1. Points of subjective equality (A) and Weber fractions (B) in five sessions (non-stimulus, 100-ms non-painful, 300-ms non-painful, 100-ms painful, 300-ms painful). The PSE and WF did not differ among these five sessions.
